# Distinct functions of mammalian RAD51 paralogs in genome maintenance

**DOI:** 10.1042/BST20250440

**Published:** 2026-09-02

**Authors:** Harsh Kumar Dwivedi, Debanjali Bhattacharya, Shariva Kadupatil, Ganesh Nagaraju

**Affiliations:** Department of Biochemistry, Indian Institute of Science, Bangalore 560012, India

**Keywords:** Cancer, DNA synthesis and repair, genome integrity, RAD51, RAD51 paralogs

## Abstract

RAD51 paralogs (RAD51B, RAD51C, RAD51D, XRCC2, and XRCC3) are evolutionarily conserved essential proteins for cell survival and genome maintenance. RAD51 paralogs were originally identified to play a role in homologous recombination-mediated repair of DNA double-strand breaks (DSBs). However, investigations over the last decade have uncovered new roles of RAD51 paralogs beyond DSB repair in replication stress responses, including replication fork progression, fork stability, and its restart. Recent structural studies have not only uncovered the molecular architecture of previously known RAD51 paralog complexes but also identified novel paralog complex assemblies, providing mechanistic insights into their various genome-maintenance functions. Additionally, a role for RAD51 paralogs in resolving R-loops has been identified, and studies with cancer-associated variants suggest that RAD51 paralogs are potential determinants of cancer susceptibility and therapeutic responses. In the present review, we highlight the recently deciphered structures and novel functions of RAD51 paralog complexes and discuss the clinical and therapeutic implications.

## Introduction

Genome integrity is essential for normal cellular function and division. Genome maintenance depends on various cellular processes, including DNA replication, cell cycle progression, and DNA repair. However, the genetic material is frequently exposed to several exogenous and endogenous stresses, which can impede ongoing replication, transcription, and other DNA metabolic processes, leading to severe consequences, such as DNA double-strand breaks (DSBs) and replication stress. During replication stress, stalled replication forks generate stretches of single-stranded DNA (ssDNA) that are rapidly coated by RPA, triggering activation of the ATR–CHK1 replication checkpoint pathway [1,2]. This checkpoint response stabilizes stalled replication forks, suppresses excessive origin firing, delays cell cycle progression, and promotes DNA repair and restart of the stalled forks to ensure completion of genome duplication [3,4]. Unrepaired DNA damage or prolonged replication stress can lead to the accumulation of mutations and chromosomal aberrations, which may ultimately result in genetic diseases and cancer [1,5–7]. Thus, cells employ numerous mechanisms to prevent genome instability, including DNA repair pathways and replication stress responses.

## RAD51 and RAD51 paralogs

RAD51 and RAD51 paralogs are an evolutionarily conserved family of ATPase proteins that have essential roles in genome maintenance, ranging from DSB repair to replication stress responses [8–10]. They were originally identified as vital homologous recombination (HR) factors that promote RAD51-mediated DSB repair. RAD51 nucleofilament formation is stimulated by RAD51 mediators, including BRCA1, BRCA2, and RAD51 paralogs [8,9,11–17].

**Figure 1:**
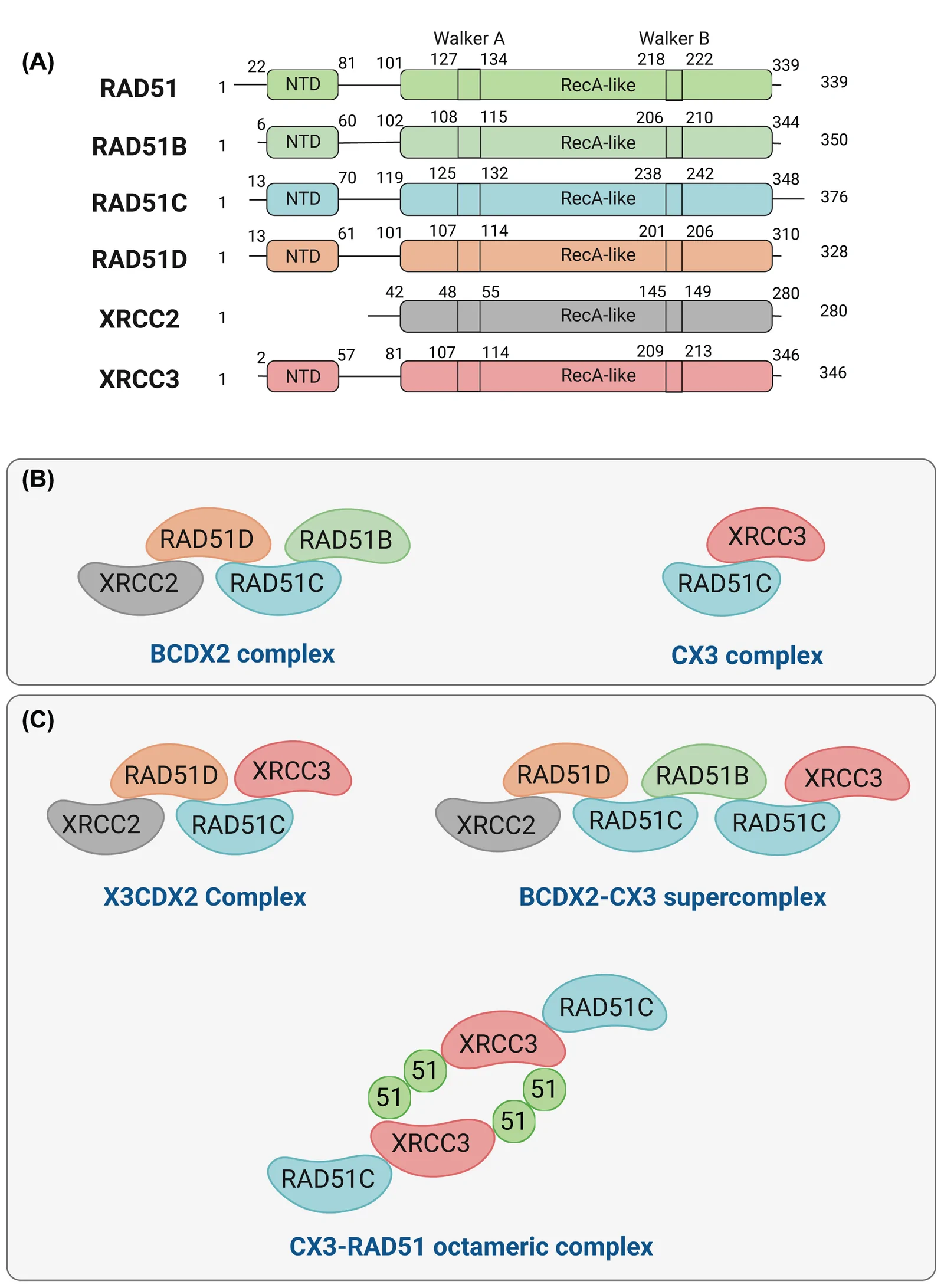
Domain architecture and distinct complexes of mammalian RAD51 paralogs (**A**) Structural architecture of RAD51 and RAD51 paralogs, highlighting the N- and C-terminal domains and their evolutionarily conserved Walker A and Walker B motifs, which are essential for ATP binding and hydrolysis. (**B**) Schematic of the two classically identified RAD51 paralog complexes: the heterotetrameric BCDX2 (RAD51B–RAD51C–RAD51D–XRCC2) complex, comprising BC and DX2 subcomplexes and the heterodimeric CX3 (RAD51C–XRCC3) complex. (**C**) Schematic of newly identified assemblies of RAD51 paralogs: the X3CDX2 (XRCC3–RAD51C–RAD51D–XRCC2) complex, the BCDX2-CX3 (RAD51B–RAD51C–RAD51D–XRCC2–RAD51C–XRCC3) supercomplex, and the octameric CX3–RAD51 complex, formed by the interaction of two tetramers (RAD51C–XRCC3–RAD51–RAD51). Created in BioRender. NAGARAJU, G. (2026) https://BioRender.com/ags4z0s.

Mammalian cells have five RAD51 paralogs: RAD51B, RAD51C, RAD51D, XRCC2, and XRCC3. RAD51 and its paralogs have conserved Walker A and Walker B motifs and exhibit ATP binding and hydrolysis (Figure 1A). RAD51 paralogs have limited sequence homology among themselves and with RAD51 which is primarily restricted to the Walker A and Walker B motifs [9,18]. Mammalian RAD51 paralogs exist in two major complexes: a heterotetrameric BCDX2 complex comprising the BC and DX2 subcomplexes and a heterodimeric CX3 complex (Figure 1B) [16,19–24]. However, recent studies identify novel assemblies of RAD51 paralogs, including a RAD51B-independent X3CDX2 complex, an octameric assembly of CX3-51, and a BCDX2–CX3-51 supercomplex (Figure 1C) [25–27]. In addition to the canonical RAD51 paralogs, a more recently identified member of the RAD51 paralog family, SWSAP1, also contains the conserved Walker A and Walker B motifs [28]. Moreover, it forms the human Shu complex along with SWS1, SPIDR, and PDS5B [29,30]. Similar to the canonical RAD51 paralogs, SWSAP1 also serves as a RAD51 mediator and facilitates RAD51 filament formation by counteracting the anti-recombinase activity of FIGNL1 [31].

Apart from their roles in DSB repair and replication stress responses, the mammalian RAD51 paralogs also participate in mitochondrial DNA replication, telomere homeostasis, and chromosome segregation [32–35]. Notably, mouse knockouts of all five RAD51 paralogs are embryonically lethal at different developmental stages, suggesting that RAD51 paralogs have non-redundant roles in genome maintenance and developmental functions [36–42]. Cells lacking RAD51 paralogs exhibit reduced HR, spontaneous genomic instability, and increased sensitivity to DNA-damaging and replication-stress-inducing agents [42–44]. Germline mutations in RAD51 paralogs cause breast, ovarian, and other cancers [45–48]. Moreover, mutations in RAD51C and XRCC2 have been reported to cause Fanconi anemia (FA)/FA-like disorder, a rare genetic disorder associated with bone marrow failure and cancer predisposition [49,50]. In the present review, we discuss the diverse functions of RAD51 paralogs in HR and replication stress responses, including a novel role in R-loop resolution and the emergence of new complexes. We also discuss potential strategies of targeting RAD51 paralogs for cancer therapy.

## RAD51 paralogs in DNA repair

DSBs are among the most lethal DNA lesions, which arise from both exogenous sources, such as radiation and chemotherapy, and endogenous sources, including reactive oxygen species, replication stress, transcription-replication conflicts (TRCs), and programmed cellular processes. Impaired DSB repair can lead to mutations, chromosomal rearrangements, various genetic disorders, and tumorigenesis. HR and non-homologous end joining (NHEJ) are the two major pathways of DSB repair. Although NHEJ is robust and active in all stages of the cell cycle, it can be error-prone due to micro-insertions or deletions at the break sites. HR, on the other hand, uses a homologous template for repair and is considered a high-fidelity pathway for DSB repair. In somatic cells, HR utilizes the neighboring sister chromatid as a template for repair and is therefore restricted to the S and G2 phases of the cell cycle [10,51,52].

**Figure 2:**
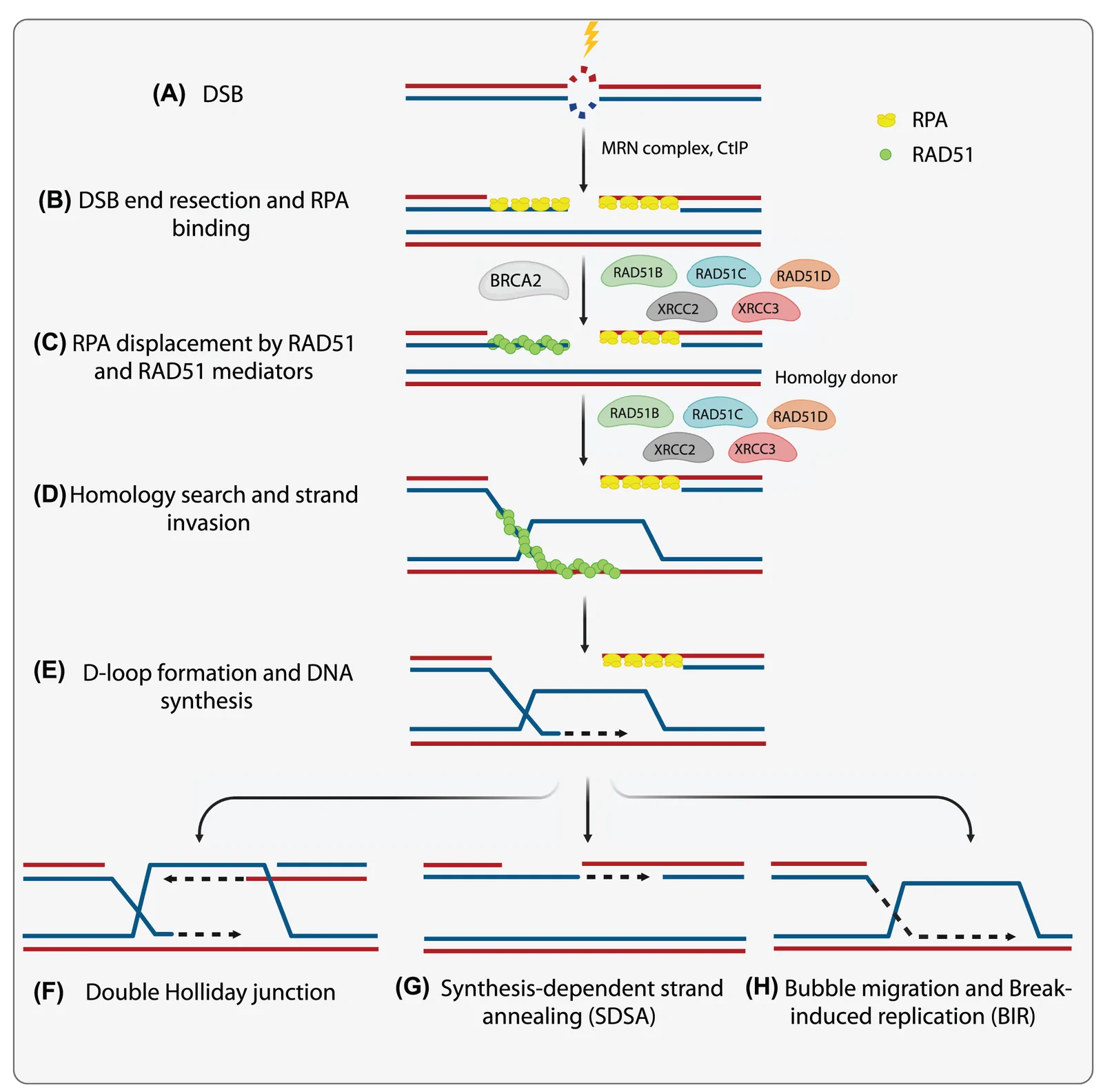
Schematic of DNA DSB repair by homologous recombination (**A**) Formation of a DNA DSB occur when both strands of DNA are incised. (**B**) DSB ends undergo resection by the action of nucleases such as the MRN complex, which creates 3’ overhangs that are rapidly coated by RPA. (**C**) RAD51 mediators, BRCA2, and RAD51 paralogs overcome RPA inhibition to promote RAD51 nucleation and filament formation onto the ssDNA. (**D**) RAD51 pre-synaptic nucleofilament perform homology search and initiates strand invasion into the homologous template. (**E**) The strand exchange activity leads to D-loop formation and the subsequent initiation of DNA synthesis. (**F**) Capture of the second end leads to the formation of a double Holliday junction, which can be either dissolved or resolved, yielding non-crossover and crossover products, respectively. (**G**) D-loop dissociation and strand annealing lead to synthesis-dependent strand annealing (SDSA) mechanism of repair, which is the predominant non-crossover outcome in mitotic cells. (**H**) Failure to capture the second end leads to break-induced replication (BIR), which involves extensive DNA synthesis by a migrating D-loop. Created in BioRender. NAGARAJU, G. (2026) https://BioRender.com/zkawcw0.

HR requires resected DNA ends on which RAD51 forms a nucleofilament and performs a homology search to initiate HR repair [53]. Formation of RAD51 pre-synaptic filaments onto the RPA-coated 3′ ssDNA overhangs is the rate-limiting step of HR-mediated DSB repair. RAD51 mediators, such as BRCA2, and RAD51 paralogs are necessary to overcome RPA inhibition and promote RAD51 nucleation and filament formation on ssDNA (Figure 2) [8,9,11,54,55]. BRCA2 localizes to DSBs through its interaction with PALB2 and mediates RAD51 binding and recruitment to ssDNA through its BRC motifs and C-terminal domain [56,57]. RAD51 paralogs are considered to facilitate several aspects of RAD51 dynamics, including filament nucleation, elongation, stabilization, and remodeling [52,58]. However, the distinction between the functions of the BRCA2 and RAD51 paralogs as RAD51 mediators during HR remains unclear.

In mammals, loss of RAD51 paralogs impairs HR efficiency with an increase in long-tract gene conversion events [59–62]. Defective HR repair confers spontaneous genomic instability and sensitivity to DNA-damaging agents and ionizing radiation in cells lacking RAD51 paralogs. Identification of RAD51C and XRCC2 as FA pathway genes demonstrated their roles in inter-strand crosslink (ICL) repair [11,49,50]. RAD51C is dispensable for ICL unhooking and FA pathway activation but is essential for its downstream role in repairing ICL-induced DSBs via HR [63]. RAD51C also participates in ICL and DSB-induced DNA damage signaling and regulates the intra-S-phase checkpoint through CHK2 activation [63,64]. Moreover, ATM/ATR-mediated phosphorylation of XRCC3 is crucial for checkpoint regulation and HR-mediated repair [65]. Although several studies have established the functions of RAD51 paralogs in HR-mediated DSB repair, the molecular mechanisms underlying their role in this process remain elusive and require further investigation.

Recent structural and single-molecule studies have revealed that while the BCDX2 complex promotes RAD51 nucleation and filament assembly [66,67], X3CDX2 complex stabilizes mature filaments by acting as a 5′ cap and prevents filament disassembly [25–27]. Furthermore, newly identified octameric CX3-RAD51 and BCDX2-CX3-RAD51 higher-order assemblies suggest that RAD51 paralogs regulate RAD51 dynamics through multiple mechanisms, including selective ssDNA engagement, ATP-dependent RAD51 loading, and homology search [25,27], warranting further studies in this direction (Figure 1C).

## RAD51 paralogs in replication stress responses

The accurate transmission of genetic information across cellular generations through DNA replication is essential for life. However, the DNA replication machinery is constantly challenged by numerous intrinsic and external sources, leading to replication stress. Replication stress threatens genomic stability, and chronic replication stress has been identified as a hallmark of cancer cells. To cope with impediments to DNA replication, cells have evolved with intricate mechanisms to sense, alleviate, and tolerate replication stress [2].

The replication stress response coordinates three major events in the cell: (i) activation of the replication checkpoint, (ii) stabilization of stalled replicating forks through fork remodeling and recruitment of essential fork protection factors, and (iii) restarting stalled forks once the stress is alleviated to ensure genome duplication [1]. Emerging studies indicate that the RAD51 paralogs contribute to several aspects of the replication stress response, beyond their role in DNA repair.

### Role in checkpoint activation and fork progression

RAD51 paralogs are abundantly present on S-phase chromatin and specifically enriched at the stalled replication sites, suggesting their involvement in replication stress responses [68]. A recent study showed that RNF20-mediated H2B monoubiquitination facilitates the loading of RAD51 and RAD51C at stalled forks through chromatin decompaction [69]. Interestingly, another study elucidated the role of G9a-mediated H3K9 trimethylation in facilitating RAD51 assembly at stalled replication sites [70]. These observations suggest that the recruitment of RAD51 and its paralogs to stalled replication forks depends on histone-modification-mediated chromatin dynamics. RAD51C/XRCC3 participates in ATM/CHK2-mediated intra-S-phase checkpoint activation in response to DSBs [63,64]. However, whether RAD51 paralogs contribute to the ATR-mediated replication checkpoint activation remains unknown. Notably, a subset of the RAD51 paralogs, the RAD51D–XRCC2 (DX2) subcomplex, has an ATR-dependent role in regulating fork progression during fluctuations of cellular dNTP pools. XRCC2 deficiency is characterized by elevated RRM2 levels, leading to increased dNTP levels and unrestrained fork progression during hydroxyurea (HU)-induced replication stress. Notably, ATR-mediated phosphorylation of XRCC2 at S247 is essential for regulating fork progression but is dispensable for HR [71]. Unrestrained fork progression in the absence of XRCC2 or defective activation of XRCC2 leads to the accumulation of ssDNA gaps and fork breakage, which promote early activation of XRCC3 by ATM/ATR-mediated phosphorylation. XRCC3 S225 phosphorylation facilitates post-replicative recombination-mediated repair of replication-born lesions and supports cell survival in XRCC2-deficient cells experiencing replication stress [71,72].

Replication forks are frequently challenged by spontaneous replication stress arising from template lesions, DNA secondary structures, DNA-bound proteins, and TRCs. Stalled forks undergo remodeling by RAD51-mediated fork reversal, which slows down DNA replication, stabilizes the stalled forks, and facilitates the restart of replication [73]. In the absence of anti-recombinase factors such as RTEL1 and RECQL5, RAD51 and RAD51 paralogs accumulate at stalled forks, leading to excessive fork reversal and hyper-recombination by RAD51, thereby impeding genome-wide replication [74,75]. These findings highlight the importance of a critical balance between HR and anti-HR activities for maintaining normal replication fork progression.

### Role in stalled fork stabilization

**Figure 3:**
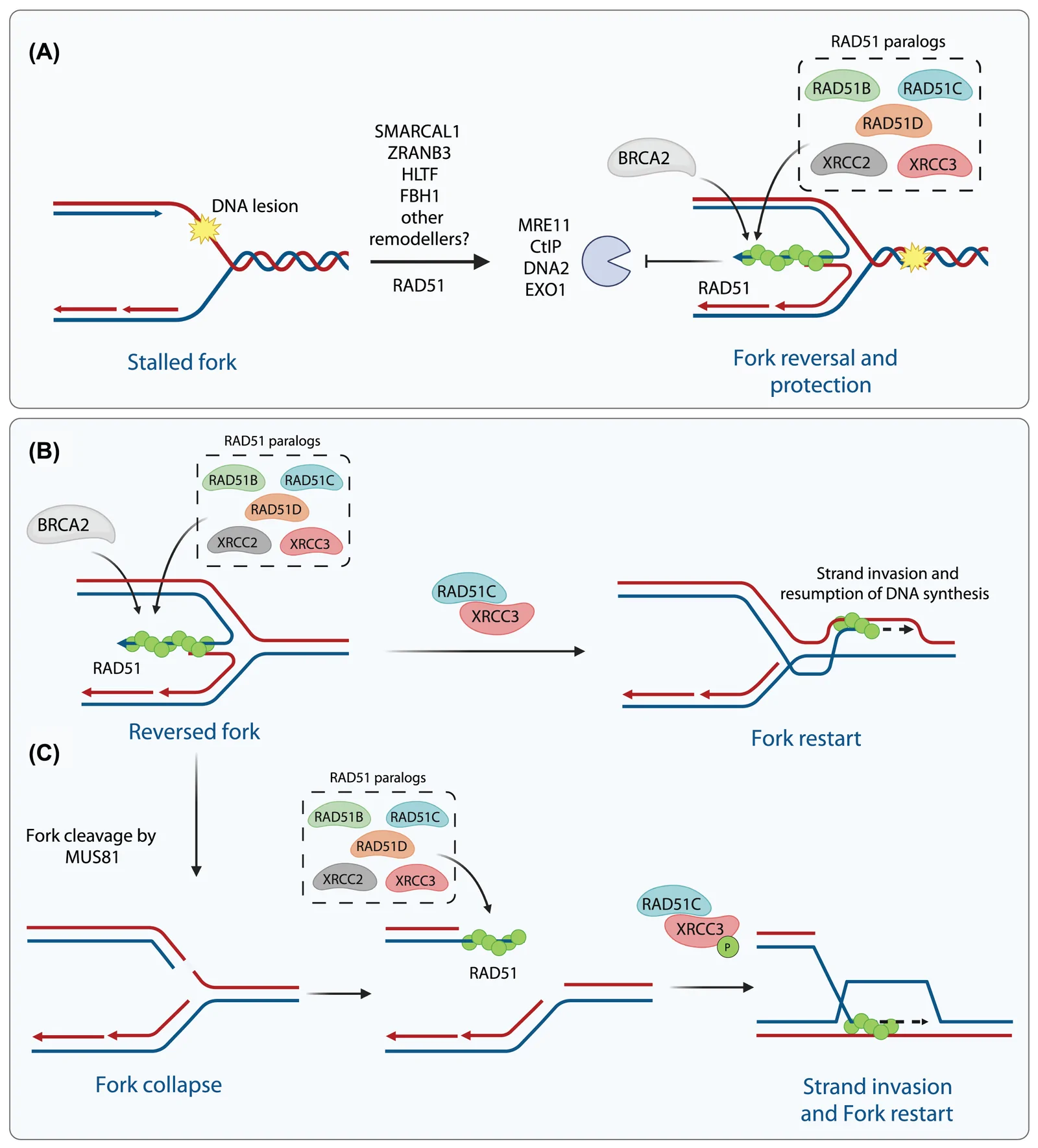
Role of RAD51 paralogs in the replication stress response (**A**) Stalled replication forks undergo fork reversal mediated by fork remodelers and RAD51. BRCA2 and RAD51 paralogs prevent nucleases from degrading reversed forks by stabilizing the RAD51 nucleofilament. (**B**) Strand invasion by the RAD51 filament facilitates restart of the stalled fork in an HR-dependent manner. The CX3 complex exclusively engages in the restart of stalled replication forks. XRCC3 phosphorylation is dispensable for stalled fork restart. (**C**) Prolonged replication stress can lead to the cleavage of reversed forks by structure-specific nucleases such as MUS81, generating one-ended DSBs. RAD51 paralogs mediate the formation of RAD51 filaments at these break ends, which facilitates fork restart through strand invasion. The CX3 complex facilitates the restart of collapsed forks in an XRCC3 phosphorylation-dependent manner at S225. Created in BioRender. NAGARAJU, G. (2026) https://BioRender.com/uga9udb.

Replication-stress-associated fork remodeling involves active backtracking and reannealing of parental DNA strands, as well as the annealing of nascent strands to form a four-way fork structure, known as the reversed fork [76]. Although largely considered a protective structure, the regressed arm of a reversed fork mimics a single-ended DSB, rendering it highly susceptible to degradation by various nucleases (Figure 3A) [77], ultimately driving chromosomal instability and cell death [78–81]. Several DNA repair proteins are essential for fork protection during replication stress.

Seminal studies from the Jasin and Venkitaraman groups showed that BRCA2 participates in fork protection in a repair-independent manner [78,82]. Subsequently, it was shown that BRCA1 and FANCD2 also participate in the stabilization of stalled replication forks [83,84]. Investigations with Chinese hamster cells deficient in RAD51C, XRCC2, and XRCC3 showed fork degradation by MRE11 nuclease, indicating that both the BCDX2 and CX3 complexes participate in fork protection during replication stress (Figure 3A) [68,69,71]. Interestingly, abrogation of ATR-mediated phosphorylation of XRCC2 at S247 enhances nascent strand degradation and leads to early XRCC3-activation during HU-induced replication stress [72]. Contrastingly, XRCC3 phosphorylation at S225 by ATR is dispensable for stalled fork protection but required for HR-mediated repair of replication-associated lesions and recovery of stalled/collapsed forks, indicating that XRCC2 and XRCC3 are distinctly activated by ATR to protect, repair, and restart the stalled/collapsed forks [65,72]. Consistent with genetic and cell biological studies, biochemical analyses with purified BCDX2 and CX3 complexes also showed that both BCDX2 and CX3 complexes synergize with RAD51 to protect stalled forks from MRE11 and EXO1 nucleases [85]. Emerging studies show that RAD51 promotes fork reversal and participates in fork protection as a global response to a wide range of replication stress-inducing agents [73,86–88]. The RAD51-mediated fork reversal occurs independently of BRCA2 [89,90] and RAD51 paralogs [85], while both are important for stalled fork protection. The RAD51 metafilaments are sufficient for promoting fork remodeling, whereas stable filaments are essential for fork stabilization [89,91]. Apart from the role of RAD51 paralogs in reversed fork stabilization, a study implicates the BCDX2 complex in promoting fork reversal [92]. The differential findings regarding the role of BCDX2 complex in fork reversal and protection may arise from different gene-silencing methods, yielding varying levels of knockdown across studies. RAD51 has been reported to function in a quantity-dependent manner, with higher cellular levels of RAD51 required for fork protection than for promoting fork reversal [91]. However, whether RAD51 paralogs function in a quantity-dependent manner, similar to RAD51, with different knockdown levels leading to distinct fates of stalled replication forks, warrants further investigation.

A recent study shows two independent pathways of fork remodeling, mediated by the SNF2-family of DNA translocases (SMARCAL1, ZRANB3, and HLTF) and the FBH1 helicase [93]. These fork remodelers function upstream of distinct sets of fork protection factors [94,95]. For example, while BRCA2 and FANCD2 protect forks remodeled by the SNF2-remodelers, FBH1-remodeled forks rely on 53BP1 and BOD1L for fork stabilization [96]. Interestingly, a recent study found that the RAD51 paralogs do not participate in the canonical SMARCAL1-BRCA2 pathway of fork stabilization [97]. This study shows that different RAD51 paralog complexes are involved in separate pathways of fork stabilization. The DX2 and CX3 complexes stabilize forks that have been remodeled by FBH1, while the BC subcomplex protects forks remodeled by the FANCM translocase [97]. Furthermore, depletion of XRCC2 and RAD51C exacerbates fork degradation in BRCA2-deficient Chinese hamster cells, indicating additional roles of these paralogs in fork protection [68]. Indeed, combining Rad51c and Brca2 homozygous hypomorphic mutants in mice phenocopies FA syndrome, characterized by bone marrow failure, rapid cancer development, and cancer-drug hypersensitivity. While single hypomorphic mutants do not lead to disease development, mouse cells harboring double mutations in Brca2 and Rad51c show severe fork protection defects at both high and low doses of HU and chromosomal instability along with compromised cell survival under mitomycin C (MMC) treatment [98]. These observations demonstrate how polygenic mutations exacerbate replication stress and highlight the contribution of secondary mutations in driving genome instability and disease.

Functional characterization of cancer-associated variants of the RAD51 paralogs has provided insights into their role in genome maintenance and preventing tumorigenesis. Somyajit et al. reported that ATP binding, but not hydrolysis, is essential for fork protection by RAD51C and XRCC3, and RAD51C L138F and R258H pathological mutants are defective for fork protection [68]. Recent advances in understanding the structure-function relationships of RAD51 paralog complexes have shed light on the replication-associated roles of RAD51C. A study by Longo et al. shows that cancer-associated RAD51C variants, such as A126T and G264S, do not exhibit an HR defect as measured by DR-GFP assays in isogenic HAP1 cell lines; however, these cells accumulate endogenous micronuclei compared with WT RAD51C cells, indicating unresolved replication stress [99]. A126T RAD51C shows fork protection defects, and the crystallographic structure identifies that A126T is in the C-terminal domain interface (CTDI) region of the CX3 complex, implying that mutations in this region result in defective ATP binding and fork protection [99]. Another study also identified several cancer-associated RAD51C mutations, which also coincide with the CTDI or its adjacent regions of the CX3 complex [100], further emphasizing the importance of ATP binding in the fork maintenance by RAD51C [99]. These studies uncouple the role of RAD51 paralogs during HR and replication stress and highlight the importance of screening for replication phenotypes of pathological RAD51 paralog mutants to better understand disease pathogenesis, drug sensitivities, and treatment strategies.

### Role in fork restart

Among the two major paralog complexes—BCDX2 and CX3—only the CX3 complex participates in restarting stalled/collapsed replication forks, marking a separation of function between the two paralog complexes (Figure 3B) [68,92,101]. Stalled forks can be restarted in an HR-dependent manner that requires homology search and strand invasion by the RAD51 nucleofilament. XRCC3 facilitates the chromatin loading of RAD51 during the restart of stalled forks generated after short HU exposure, implicating XRCC3 function in RAD51-mediated fork recovery (Figure 3B) [65]. The structural conformation of the CX3/X3CDX2 complex promotes capping of the RAD51 filament at the 5′ ends, stabilizing and promoting its strand exchange activity [26,99]. Consistent with this role, the CX3 complex may facilitate strand invasion of the RAD51-coated reversed fork end into the parental duplex ahead of the fork junction, thereby resetting the reversed fork and promoting fork restart. However, considering recent studies [25–27], it remains to be investigated whether only the CX3 complex or the X3CDX2 complex is involved in the restart of the stalled fork.

Prolonged replication stress can lead to cleavage of the stalled fork by structure-specific nucleases such as MUS81 or SLX4, generating collapsed forks with a one-ended DSB [77,102]. While ATM/ATR-dependent phosphorylation of XRCC3 at S225 is dispensable for stalled fork restart, it is essential for the recovery of collapsed forks, as XRCC3 phosphodeficient cells display inefficient chromatin loading of RAD51 following prolonged HU treatment (Figure 3C) [65,72]. Thus, ATM/ATR-mediated phosphorylation of XRCC3 distinguishes its role in stalled and collapsed fork recovery.

The fork restart function of the CX3 complex is dependent on ATP-binding and ATP hydrolysis, as both ATP-binding and hydrolysis-defective mutants of RAD51C and XRCC3 display defects in fork restart [68,103]. Compared with wild-type (WT) cells, ATPase-mutants of both RAD51C and XRCC3 fail to disengage RAD51 from the fork sites during recovery from replication stress, indicating that, after strand exchange, the ATPase activity of CX3 facilitates RAD51 disassembly from replication forks to promote fork restart [68].

Recent studies have identified fork restart defects in several cancer-associated RAD51C variants. Longo et al. find that G264S RAD51C is defective for fork restart and identify a previously uncharacterized DNA-binding region in the N-terminal domain interface (NTDI) between RAD51C and XRCC3, which is required for ATP hydrolysis and fork restart functions [99]. This correlates with another study showing defective fork restart in R258H RAD51C-expressing cells [68], as the R258 residue also lies within the NTDI region. Interestingly, both R258H and G264S RAD51C mutations have been identified in patients with breast cancer and FA-like disorders, highlighting a possible link between fork restart defects leading to cancer-predisposition diseases [98]. Notably, although BRCA2 is essential for fork protection, it is dispensable for the restart of stalled forks [89,90]. Indeed, mouse cells having hypomorphic mutation in Brca2 do not exhibit replication restart defect and these cells with combined hypomorphic Rad51c mutation show a severe fork restart defect [98], highlighting the role of RAD51C in fork restart and genome stability. It will be interesting to investigate whether RAD51 paralogs facilitate stalled/collapsed fork restart through their interactions with DNA helicases/translocases or with other factors.

### Emerging role in R-loop resolution

R loops are three-stranded structures consisting of an RNA-DNA hybrid and a displaced ssDNA strand [104]. These structures serve as crucial intermediates in several physiological processes, including transcription, DNA replication, and recombination-mediated repair of DSBs [104,105]. However, aberrant accumulation or persistence of R-loops can lead to genome instability, underscoring the importance of regulating R-loop levels in cells [104,105]. Numerous factors, including HR- and FA-pathway proteins such as BRCA1, BRCA2, FANCA, FANCD2, and FANCM, are involved in suppressing R-loops and associated genome instability [106–112].

**Figure 4:**
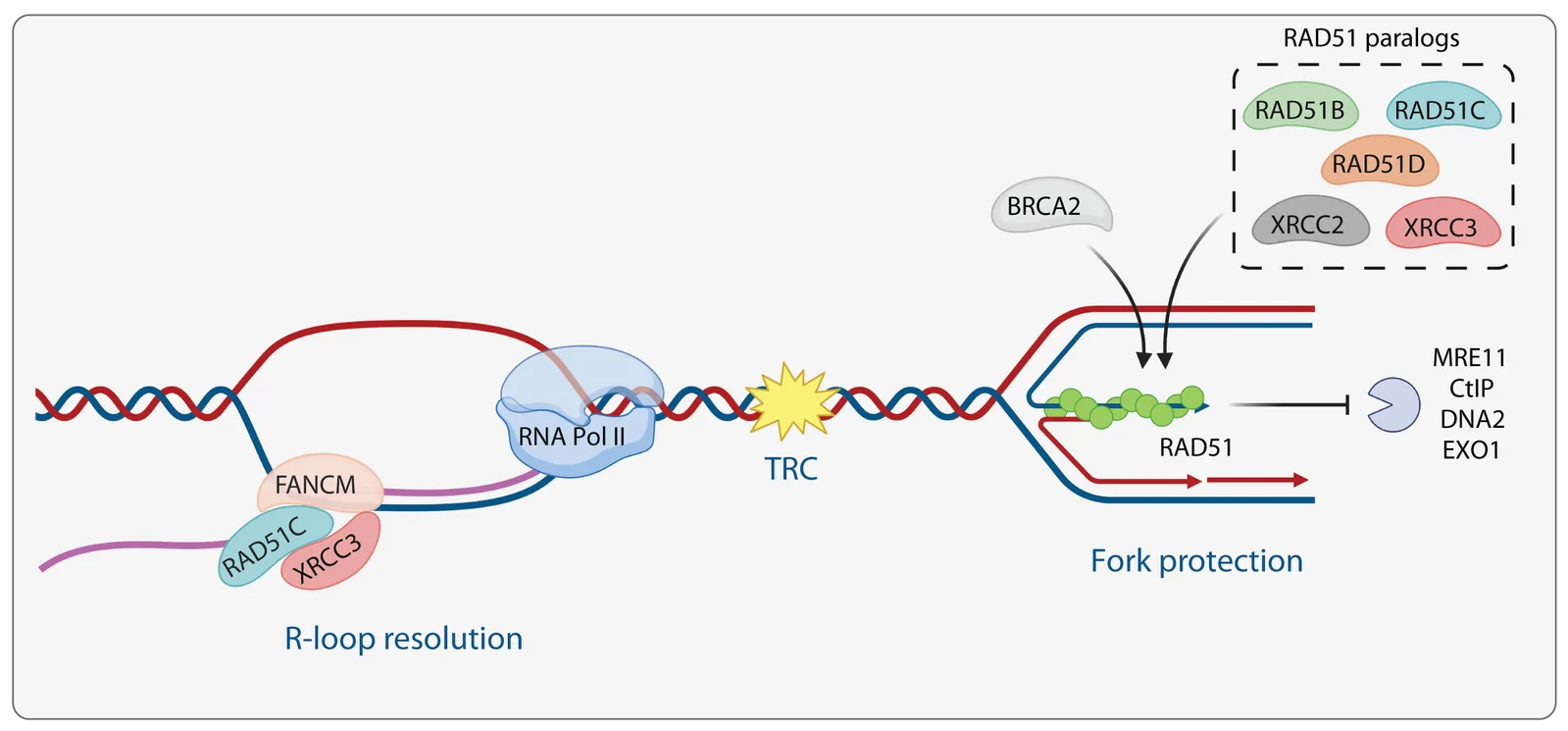
Emerging role of the CX3 complex in R-loop resolution The CX3 complex physically interacts with the FANCM translocase to facilitate its recruitment to R-loop sites and promote R-loop resolution. The role of the CX3 complex in R-loop resolution is independent of its replication fork-associated functions. Created in BioRender. NAGARAJU, G. (2026) https://BioRender.com/4vv17kc.

A recent study by Sahoo et al. identified the role of the RAD51 paralog complex CX3 in preventing R-loop accumulation [103]. Mechanistically, the CX3 complex participates in the FA pathway of R-loop resolution. The CX3 complex physically interacts with the FANCM translocase and facilitates its recruitment to R-loop sites for their resolution (Figure 4). Interestingly, the RAD51C R258H pathological mutant, identified in FA-like disorder, is defective in its interaction with FANCM and exhibits impaired resolution of R-loops/TRCs [103]. Notably, the RAD51C ATPase mutants that are defective for fork maintenance functions are competent to interact with FANCM and promote R-loop resolution similar to WT RAD51C, suggesting that CX3 complex-mediated R-loops/TRCs resolution is independent of its fork-associated functions [103]. These findings indicate that the CX3 complex acts as an adaptor for FANCM recruitment to R-loop sites and its resolution through FANCM-mediated translocase activity [103]. However, whether the CX3 complex can directly localize to R-loop sites, or whether its binding to R-loops is facilitated by chromatin remodeling or other factors, remains to be investigated. Moreover, whether the CX3 complex interaction with FANCM stimulates its R-loop resolution activity needs further investigation.

## Structural features of RAD51 paralogs

Cryo-EM structure of the human BCDX2 complex reveals extensive intermolecular interactions between the NTDs and ATPase domains of the subunits, stabilizing the tetrameric complex. The NTDs of RAD51B, RAD51C, and RAD51D form a tripartite stacking interaction network at the periphery of the complex [66,67]. Biochemical and biophysical studies show that BCDX2 binds to the RPA–ssDNA complex and facilitates RAD51 nucleation and extension in an ATP-dependent manner. The RAD51–ssDNA filament assembly by BCDX2 is mediated by its interaction with RAD51 and ssDNA; however, BCDX2 does not interact directly with RPA and selectively binds to the ssDNA gaps within RPA-coated ssDNA substrates [66,67]. Due to its high DNA-stimulated ATPase activity, BCDX2 dissociates from ssDNA post RAD51 nucleation and does not remain associated with mature RAD51 filaments [66,67].

X-ray crystallographic structure of the CX3 complex from the extremophile metazoan *Alvinella pompejana* reveals two new interaction interfaces between RAD51C and XRCC3, namely NTDI and CTDI, where the NTD and CTD of XRCC3 interact with the C-terminal ATPase domain of RAD51C, respectively [99]. The study reveals that RAD51C variant clusters within the newly described CX3 interfaces exhibit functional defects in replication stress responses [99].

Insights from the engagement of RAD51B and XRCC3 at a common interface on RAD51C and the presence of an unoccupied NTD on RAD51C in the CX3 complex led to the identification of a novel X3CDX2 complex (Figure 1C) [25–27]. Cryo-EM, along with single-molecule and biochemical investigations, revealed that the X3CDX2 complex promotes pre-synaptic filament assembly by facilitating RAD51 nucleation on ssDNA and stimulates RAD51-mediated strand invasion [25–27]. Moreover, the X3CDX2 complex also caps the RAD51 nucleofilament at the 5′ end, with XRCC3 directly interacting with RAD51 and XRCC2 forming the cap [25–27]. Unlike the BCDX2 complex, which shows a high rate of ATP hydrolysis-mediated disassembly from the ssDNA due to coupled ATPase activity of RAD51B and RAD51C, the X3CDX2 complex shows minimal ATPase activity and remains stably associated with the ssDNA [26]. Due to its minimal ATPase activity, the X3CDX2 complex stably anchors and stabilizes the RAD51 filament on ssDNA, possibly preventing filament disassembly through the activities of anti-recombinases such as RECQL5, FIGNL1, and PARI, and potentially facilitating rapid re-nucleation after disassembly [25–27,113–116].

A recent cryo-EM study reports an octameric CX3-RAD51 assembly formed by the interaction of two tetrameric (RAD51–RAD51–XRCC3–RAD51C) protomers (Figure 1C) [27]. The complex is uniquely stabilized by a loop insertion from XRCC3 into the terminal RAD51 of the neighboring tetramer and an interaction of the FXXA motif of RAD51 with the hydrophobic pocket in XRCC3 [27]. The octameric complex prevents nonproductive binding of RAD51 to dsDNA or RNA–DNA hybrids while preserving its binding to ssDNA and facilitates RAD51-mediated DNA strand exchange [27].

The formation of the X3CDX2 complex is also consistent with the existence of the octameric CX3–RAD51 complex. The binding of DX2 to the exposed RAD51C of the CX3–RAD51 complex disrupts the stabilizing interactions of the octameric complex and forms a pentameric 51–X3CDX2 complex [27]. The X3CDX2 complex, unlike the BCDX2 complex, is unable to stimulate RAD51 filament formation on RPA-coated ssDNA [26,67]. Moreover, the X3CDX2 complex, but not the BCDX2 complex, facilitates RAD51-mediated strand exchange and promotes D-loop formation in the absence of RPA [26]. However, whether the X3CDX2 complex can overcome the RPA inhibition remains unclear and requires further investigation. Furthermore, the role of the X3CDX2 complex in mediating RAD51 filament stability and strand exchange can be inferred in the context of replication stress. Although knockout of all five Rad51 paralogs causes embryonic lethality in mice, RAD51B deficiency is better tolerated than the other paralogs in human cell lines with isogenic disruption mutants of the RAD51 paralogs [42]. The higher sensitivity of RAD51C-, RAD51D-, XRCC2-, and XRCC3-deficient cells to MMC or olaparib suggests crucial roles for the X3CDX2 complex in stalled fork stabilization and fork restart [26], although the mechanistic distinctions in X3CDX2 functions in these processes remain to be explored.

Another recent cryo-EM study reports that all five RAD51 paralogs can form a higher-order assembly in an ATP-dependent manner, resulting in a BCDX2–CX3-51 supercomplex (Figure 1C) [25]. The supercomplex mimics a RAD51 filament on the ssDNA with CX3 stacking directly over BCDX2, creating a platform for RAD51 loading. The DX2 module ensures ssDNA selectivity, and the CX3 module splits the ssDNA into nucleotide triplets, probably facilitating homology search. The BCDX2–CX3 complex bound to RAD51 exhibits high ATPase activity in the presence of ssDNA, suggesting a highly dynamic assembly and ATP-hydrolysis-mediated disassembly [25]. Overall, these cryo-EM and X-ray crystallography structures highlight the modular nature and functional specialization of distinct RAD51 paralog complexes.

## RAD51 paralogs mutations and cancer

Mutations in HR genes are prevalent across a variety of cancers, including breast and ovarian cancers. *BRCA1* and *BRCA2* are the most frequently mutated HR genes in cancers; however, several studies report that germline mutations in other HR genes, including *RAD51* paralogs, are also associated with cancer predisposition [45–48,117–122]. HR-deficient cancers are clinically targeted with chemotherapeutic drugs, such as poly(ADP-ribose) polymerase (PARP) inhibitors [123]. However, a systematic clinical classification of the pathogenicity associated with HR gene variants is essential for accurate cancer risk prediction and the efficacy of targeted therapies. Despite being included in hereditary cancer screening panels, most of the RAD51 paralog mutations identified in cancers were classified as variants of unknown significance (VUS).

Several recent studies have attempted to experimentally determine the pathogenic status of RAD51 paralog variants. Studies characterizing pathological mutants of RAD51 paralogs reveal that cancer-associated RAD51D variants (G96C and G107V) that disrupt its interaction with XRCC2 exhibit defective HR repair [121]. Multiple studies pertaining to RAD51C variants indicate that missense mutations in or near the Walker A and Walker B motifs are HR-defective [124,125]. Moreover, mutations in RAD51C residues that are conserved with RAD51 are often associated with severe HR defects [124]. Notably, previous studies with RAD51C pathological mutants showed that mutations in residues conserved between RAD51C and RAD51, such as G125V and L138F, exhibit more severe HR defects and greater sensitivity to ICL-inducing agents than mutations in non-conserved residues [63]. Interestingly, Walker B motif ovarian cancer variants L238R and V239G of RAD51C also exhibit separation of function between HR and replication, with L238R being proficient for replication roles and defective for HR, and V239G being HR-proficient and defective for replication functions [125]. A recent saturation genome editing (SGE) assay using cell fitness as the primary readout has provided functional defects associated with all possible single-nucleotide variants and a spectrum of multiple-nucleotide variants of RAD51C [126]. SGE analysis revealed that variants at evolutionarily conserved residues exhibit more severe defects in cellular fitness than those at less evolutionarily conserved residues. This finding suggests that mutations at less evolutionarily conserved residues are hypomorphic [126]. Interestingly, mutations in the RAD51C residues critical for the assembly of the octameric 51C–X3 complex and the X3CDX2 complex are also associated with reduced cellular fitness in the SGE analysis, indicating the critical role of these residues in RAD51C functions, RAD51 paralog complex assembly, and cancer risk [27]. Furthermore, several residues recently identified as critical for DNA binding and paralog–paralog interactions within RAD51 paralog complexes were previously classified as VUS [26,67]. These findings underscore the functional relevance of these variants and suggest that the phenotypes associated with them likely arise from the disruption of interactions mediated by these residues.

Overall, characterizing cancer-associated variants and including RAD51 paralogs in HR-deficiency screens in patients will provide better therapeutic strategies and aid precision medicine approaches in the future. Furthermore, the clinical classification of variants primarily focuses on HR-related functions of RAD51 paralogs to determine pathogenicity; however, analyses of replication-associated functions would also provide deeper insights into the onset of the disease and therapeutic interventions.

## Targeting RAD51 paralogs for cancer therapy

Cancer cells depend on replication stress response pathways for survival and proliferation [77]. Several chemotherapeutic drugs, including PARP inhibitors, exploit replication stress as a vulnerability to specifically eliminate cancer cells [123]. Considering the role of RAD51 paralogs in replication stress response pathways and mediating RAD51 activity [9], disrupting RAD51 paralog functions with small-molecule inhibitors could increase replication stress and DNA damage burden in cancer cells, which could be exploited for targeted therapy in a clinical setting.

HR and fork protection defects in BRCA-mutated cancers are exploited to selectively target these cancers with PARP inhibitors. Although PARP inhibitors effectively target BRCA-deficient cancers, many patients eventually develop resistance [123,127,128]. Several resistance mechanisms have been identified that restore HR and fork protection defects in these cancers, including reversion mutations in BRCA1/2, loss of NHEJ promoters such as 53BP1, Shieldin complex, HELB, DYNLL1, and ERCC6L2, and elevated RAD51 protein levels [129–135]. Since RAD51 paralogs play crucial roles in both HR repair and fork protection, they could be targeted to resensitize PARP inhibitor-resistant cancers.

RAD51 and RAD51 paralogs are overexpressed in many types of cancer [9,129,136]. Recent structural studies and the emergence of novel RAD51 paralog complexes will provide crucial insights into the development of small-molecule inhibitor drugs. Small-molecule inhibitors could be designed to target specific paralog-complex interfaces, ATP- and DNA-binding sites, or to disrupt RAD51 interactions, which may serve as a valuable approach in cancer therapeutics.

Patient-derived xenograft studies of high-grade serous ovarian carcinoma report that RAD51C promoter methylation leads to gene silencing and HR deficiency, rendering the cancer sensitive to PARPi therapy [137]. Moreover, loss of promoter methylation even in a single gene copy confers therapy resistance, suggesting that the methylation status of HR-related gene promoters could be explored as an indicator of PARPi therapy outcome [137,138]. However, most clinical studies on variant analysis and promoter methylation mainly focus on RAD51C. Such studies involving other RAD51 paralogs can also provide valuable insights into cancer risk prediction and clinical management of variant carriers.

## Conclusions and future directions

RAD51 paralogs maintain genome stability by mediating RAD51 functions during HR and fork protection. RAD51 paralogs have also evolved with RAD51-independent functions in modulating replication fork progression and R-loop resolution. Recent structural, single-molecule, and biophysical studies provide crucial insights into the roles of paralog complexes in DNA repair and replication. Nonetheless, further investigations are required to elucidate the precise mechanisms of their function in genome maintenance and tumor suppression.

A recent study reported that the loss of an anti-recombinase FIGNL1 can restore HR defects in BRCA2-deficient cells, thereby conferring chemoresistance to PARP inhibitors and platinum-based drugs [114,139]. Findings from the study indicated that RAD51 loading in cells deficient in BRCA2 and FIGNL1 is facilitated by the MMS22L–TONSL complex, thereby attributing HR defects in BRCA2-deficient cells to unregulated RAD51 removal by FIGNL1 rather than defective RAD51 loading, as previously believed [139]. However, the role of RAD51 paralogs in mediating RAD51 loading and stabilization in BRCA2- and FIGNL1-deficient cells remains unknown. Mechanistic insights into the interplay between RAD51 paralogs and anti-recombinases, including FIGNL1, RTEL1, RECQL5, and PARI during RAD51 loading, especially in the context of BRCA deficiency, can shed light on re-sensitization strategies for chemoresistant cancers.

Although the role of RAD51 in HR-mediated DNA repair, replication fork reversal, and protection is well established, several recent studies have identified novel roles for RAD51 in genome maintenance. RAD51 has been identified to protect under-replicated DNA by promoting mitotic DNA synthesis (MiDAS) in a polo-like kinase-dependent manner [140]. Another study has identified a novel function of RAD51 in preventing re-replication of DNA. RAD51 impedes fork progression of re-replication forks, leading to frequent fork reversal and ssDNA gap formation by PRIMPOL-mediated repriming. The ssDNA gaps are degraded by MRE11, restricting over-replication [141,142]. A recent study also shows that RAD51 specifically recognizes and binds to abasic sites, preventing fork breakage by suppressing MRE11-mediated cleavage [142,143]. These studies underscore the emerging significance of RAD51 in genome maintenance beyond its canonical functions. It would be intriguing to investigate whether RAD51 paralogs are involved in mediating RAD51 functions during MiDAS, preventing re-replication, or protecting abasic sites.

Defects in HR and fork protection in BRCA-deficient cancers have been attributed to their sensitivity to PARP inhibitors [123]. However, recent evidence implicates replicative gaps as a key determinant of PARPi sensitivity [144–148]. RAD51 participates in replication gap suppression and post-replicative gap repair [79,149,150]. In addition, RAD51 is chromatin-enriched in BRCA1-deficient cells, suggesting that inhibition of RAD51 in cancers with replicative gap vulnerabilities could have therapeutic implications [151]. Whether RAD51 paralogs function in gap suppression remains to be investigated.

## Perspectives

- RAD51 paralogs maintain genome stability by mediating RAD51 filament formation during HR-mediated DNA repair and replication stress responses.
- Recent cryo-EM studies have identified novel paralog complexes, including the X3CDX2 complex, the BCDX2–CX3-51 supercomplex, and the octameric CX3-51 complex. A novel role of the CX3 complex has also been identified in regulating R-loop resolution in a FANCM-dependent manner, highlighting an expanded role for HR factors, including RAD51 paralogs, in the replication stress response.
- RAD51 paralogs could play crucial roles in mediating novel RAD51 functions during mitotic DNA synthesis, regulating re-replication, and protecting abasic sites. In addition, RAD51 paralogs could be potentially targeted to resensitize PARP inhibitor-resistant cancers.

## Open Access

This article has been published open access under our Subscribe to Open programme, made possible through the support of our subscribing institutions, learn more here: https://portlandpress.com/pages/open_access_options_and_prices#conditional.

## Abbreviations

CTDI: C-terminal domain interface
DSB: double-strand break
FA: Fanconi anemia
HR: homologous recombination
ICL: inter-strand crosslink
MiDAS: mitotic DNA synthesis
MMC: mitomycin C
NHEJ: non-homologous end joining
NTDI: N-terminal domain interface
SGE: saturation genome editing
ssDNA: single-stranded DNA
TRC: transcription-replication conflicts
VUS: variant of unknown significance
WT: wild-type
